# Efficacy and Safety of Crizotinib among Chinese EML4-ALK-Positive, Advanced-Stage Non-Small Cell Lung Cancer Patients

**DOI:** 10.1371/journal.pone.0114008

**Published:** 2014-12-12

**Authors:** Yabing Cao, Guangli Xiao, Xibin Qiu, Sheng Ye, Tongyu Lin

**Affiliations:** 1 Department of Oncology, Kiang Wu Hospital, Estrada do Repouso, Macau, China, 999999; 2 Department of Oncology, The First Affiliated Hospital of Sun Yat-sen University, 58 Zhongshan 2nd Road, Guangzhou, Guangdong, P.R. China, 510080; 3 Cancer Center of Sun Yat-sen University, State Key Laboratory of Oncology in Southern China, Department of Medical Oncology, No. 651 Dongfeng Road East, Guangzhou, Guangdong, P.R. China, 510060; Istituto dei tumori Fondazione Pascale, Italy

## Abstract

**Introduction:**

We report the efficacy and safety of crizotinib treatment among Chinese patients with advanced-stage NSCLC.

**Methods:**

We retrospectively analyzed patients with EML4-ALK positive advanced NSCLC who were treated with crizotinib from May 2012 to Aug 2013. Baseline clinical parameters, treatment protocol, response to therapy and survival were noted. The primary goal was to evaluate the efficacy of crizotinib in patients who were previously treated patients or who had poor ECOG performance status (PS).

**Results:**

Forty patients were evaluable for safety and efficacy. Median age was 43 years, 100% had adenocarcinoma and stage IV disease, and 42.5% were female. Six patients received frontline treatment with crizotinib, 17 patients had 1 prior treatment, and 17 patients had more than 2 lines of prior treatment. Patients received a median of 5 cycles of treatment (range 1–15 cycles). After the first cycle, 92.5% (37/40) patients archived partial remission (PR). At the end of the follow-up period, the overall PR rate was 70% (28/40), and progression of disease (PD) occurred in 30% of patients (12/40). The median PFS was 28 weeks (95% CI 15.4 to 40.5 weeks), and median OS was 40 weeks (95% CI 38.6 to 49.3 weeks). The most frequent treatment-related AEs were vomiting (47.5%), vision disorder (27.5%) and increased ALT/AST (42%); most toxicities were Grade 1/2. Observed treatment-related Grade 3/4 AEs included increased ALT/AST (10%) and vomiting (5%). The EML4-ALK fusion rate and number of prior chemotherapy cycles did not appear to significantly affect the efficacy of crizotinib. However, PS 0–2 patients had improved PFS (50 weeks vs. 24 weeks, p = 0.015).

**Conclusions:**

Crizotinib was safe, well-tolerated, and effective in Chinese patients with pre-treated ALK-rearranged NSCLC. QOL was improved and PS appears to have an effect on the efficacy of crizotinib, but prior treatment and ALK fusion rate do not.

## Introduction

More than 50% of patients with non-small cell lung cancer (NSCLC) and known oncogene mutations are candidates for personalized or targeted therapy. ALK is a recently identified tyrosine kinase target in NSCLC [1]. ALK is aberrantly activated by chromosomal rearrangement or inversion that leads to expression of an oncogenic fusion kinase, such as EML4–ALK. The ALK fusion gene has been found in approximately 5% of Caucasian NSCLC patients and occurs in 3.3–6.1% of Chinese patients [2]–[4]. ALK and EGFR are generally mutually exclusive, making it a potential target for treatment.

Crizotinib is an oral tyrosine kinase inhibitor that targets ALK, MET and ROS1. Preclinical work demonstrated that cancer cells harboring EML4–ALK were highly sensitive to ALK inhibition [5]. Several phase I and II clinical trials have demonstrated the efficacy of crizotinib in advanced-stage, ALK-positive NSCLC patients, resulting in the accelerated approval of crizotinib by the FDA in August 2011[6], [7]. In the recently published PROFILE 1007 study, 159 previously treated patients were randomized to receive crizotinib or chemotherapy with pemetrexed or docetaxel until disease progression. Crizotinib had a significantly longer progression-free survival (7.7 vs. 3.0 months) and a higher overall response rate (65.3% vs. 19.5%) than chemotherapy [8]. Previous studies included only a small number of Asian patients and only enrolled patients with good PS (ECOG 0–2). The efficacy of crizotinib in Chinese patients and in patients with PS >3 was therefore unknown.

In this study, we prospectively reviewed clinical outcomes of Chinese NSCLC patients treated with crizotinib in our center and generated further efficacy and safety data in these patients.

## Methods

### Patients and treatment

Forty patients with ALK-positive, advanced-stage NSCLC who received crizotinib in our center from May 2012 to Sep 2013 were retrospectively reviewed. For all patients, ALK positivity was confirmed locally by fluorescence in-situ hybridization (FISH) or PCR using the initial diagnostic or surgical specimen. Patients who had received previous chemotherapy or EGFR TKIs were eligible. Patients received crizotinib at a dose of 250/200 mg twice daily.

### Efficacy assessments

Tumor assessments were performed before crizotinib treatment, 4 weeks after the first cycle, and every 8 weeks thereafter. The primary endpoint was objective response [complete response (CR), partial response (PR) and stable disease (SD)] as determined by RECIST version 1.0. Secondary outcome measures included progression-free survival (PFS), overall survival (OS), and duration of objective response. Quality of life (QOL) was measured using the European Organization for Research and Treatment-Quality of Life Questionnaire (EORTC QLQ-C30).

### Safety and tolerability assessments

The incidence and severity of AEs were graded according to the National Cancer Institute's Common Terminology Criteria for Adverse Events (CTCAE) version 3.0. Physical examination findings, vital signs, and laboratory studies were regularly monitored.

### Statistical analyses

The analyses in this study were descriptive and exploratory. The efficacy analysis included all patients who received at least one cycle of crizotinib. Objective response analyses were based on all patients with evaluable tumor measurements. PFS, OS, and duration of response were estimated from Kaplan–Meier curves. The safety analysis included all patients who finished at least one treatment cycle.

### Ethics Statement

The study was approved by the ethics committee of Kiang Wu hospital. All patients provided written informed consent.

## Results

Baseline demographics for treated patients are summarized in table 1. Among the 40 patients included in this report, 24 (60%) patients had an ECOG performance status (PS) of 0–2, and 16 (40%) patients had PS of 3 or greater. All the patients had adenocarcinoma that was negative for EGFR mutations. The majority (85%) had received previous chemotherapy, including platinum doublet regimens. The median number of previous chemotherapy regimens was two. The best response to chemotherapy was PR.

**Table 1:** Baseline demographics of 40 Chinese patients treated with crizotinib.

	Crizotinib N(%)
Age (years), median (range)	42(33–68)
Sex (male/female)	23/17
ECOG performance status, n (%)	
0–2	24 (60)
>3	16 (40)
Number of prior chemotherapy regimen	
1	6 (15)
2	17 (42.5)
>3	17 (42.5)
Pathology subtype	
Adenocarcinoma	40 (100)
EGFR status	
Wild type	0

For the 40 patients who received at least one cycle of crizotinib, the median duration of treatment was 5 cycles (range 1–15 cycles). Treatment was discontinued for disease progression in 70% of patients; other reasons for treatment discontinuation included AEs, financial problems and unknown reasons. Three patients required dose reduction due to side effects.

**Figure 1:**
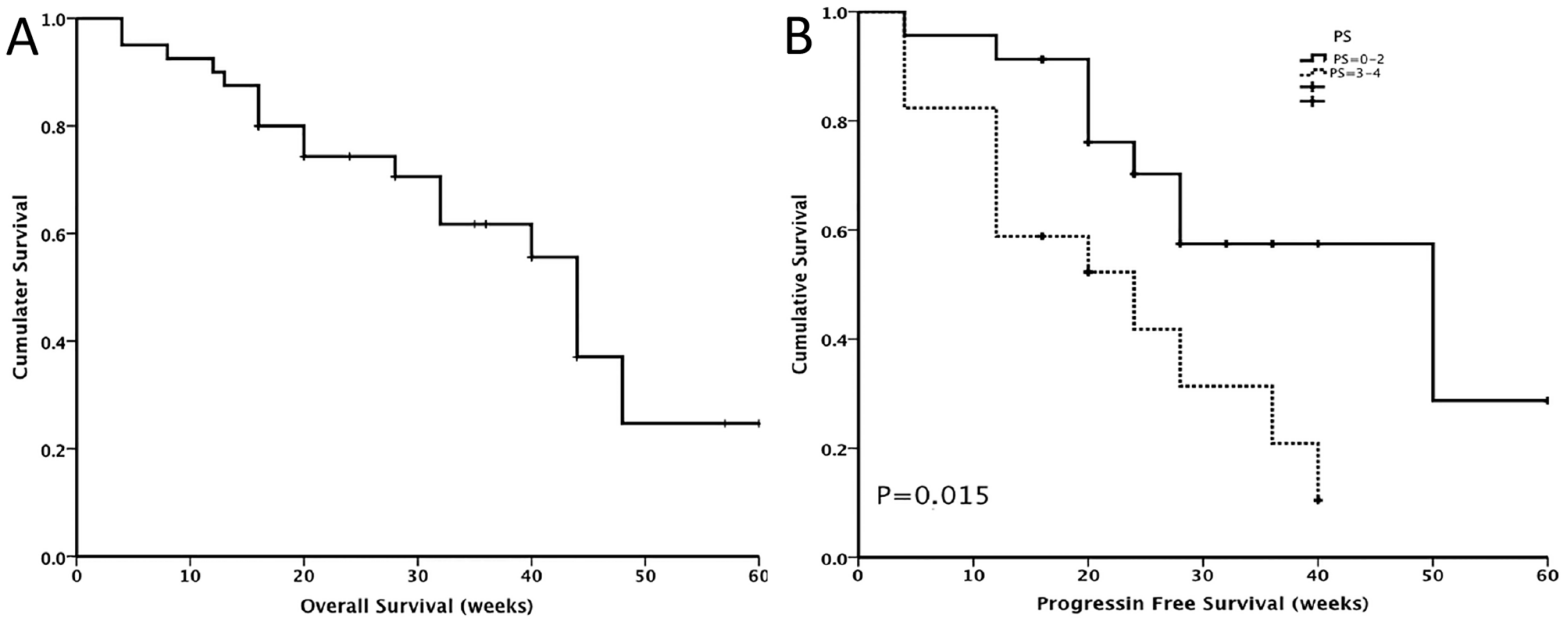
**A.** Overall of 40 Chinese patients treated with crizotinib. **B**. Progression free survival by different PS subgroup of 40 Chinese patients treated with crizotinib. PS, ECOG (Eastern Cooperative Oncology Group) performance status.

Overall, 37 of 40 (92.5%) patients achieved a PR after the first cycle of treatment (table 2). No CRs were documented. At the end of study follow-up, 28 patients (70%) were classified as having achieved clinical benefit (PR or SD) with a median duration of clinical benefit of 24 (4–60) weeks. One patient, a 48-year-old man, maintained a PR for 60 weeks and is still on treatment. A total of 12 patients had disease progression, including 9 cases of brain metastases. Seventeen patients died during or after the study. The median OS was 40 weeks (95% CI 38.6 to 49.3 weeks). (Fig. 1A)

**Table 2:** Efficacy of 40 Chinese patients treated with crizotinib according to RECIST criteria.

	Crizotinib	Duration
Treatment (cycles), median (range)	5 (1–15)	-
Efficacy		
PR after 1^st^ cycle	37 (92.5%)	-
Overall PR/SD	28 (70%)	24 (4–60) weeks
PD	12 (30%)	-

PR: partial response; SD: stable disease; PD: progressive disease

Patients with baseline good (0–2) PS had a better PFS then those with poor (>3) PS (50 weeks vs. 24 weeks, p = 0.015). (Fig. 1B) Other factors, including previous treatment received, did not appear to affect the efficacy of crizotinib (data not shown).

All patients had improvement in QOL due to relief of lung cancer-related symptoms. These symptoms include pain, dyspnea, cough, fever, anorexia and fatigue. Most patients improved during the first cycle of treatment. Patients with poor PS and short life expectancy also derived a benefit in terms of symptom relief.

Crizotinib was well tolerated (table 3). The most common AEs observed with crizotinib were vomiting (47.5%), elevated liver transaminases (40%), vision disorders (27.5%), and diarrhea (5%). The most common Grade 3/4 AEs associated with crizotinib included elevated transaminases (10%) and vomiting (5%). Four patients experienced grade 3 increases in ALT/AST, which occurred in the first cycle of treatment. Two of these patients were treated with a reduced crizotinib dose of 250 mg daily (from 250 mg twice daily), without other treatment, until normal ALT/AST levels were reached. Two patients stopped Crizotinib until ALT normalized. The duration of reduced-dose crizotinib or treatment break was 2–3 weeks. One patient treated with full-dose crizotinib (250 mg bid) complained of continuous vomiting (2–3 episodes every day in the first week), which did not respond to treatment with a 5-HT3 antagonist. When the dose was reduced to 250 mg once daily, she stopped vomiting. She tolerated full-dose treatment well one week later, when she received additional antiemetic therapy with aprepitant.

**Table 3:** Common side effects of 40 Chinese patients treated with crizotinib.

Side effects	All, N (%)	Grade 1–2, N (%)	Grade 3, N (%)
Vomiting	19 (47.5)	17(42.5)	2 (5)
Nausea	12 (30)	12 (30)	0
Diarrhea	13 (32.5)	13 (32.5)	0
Elevated ALT/AST	16 (40)	12 (30)	4 (10)
Vision disorder	11 (27.5)	11 (27.5)	0
Fatigue	4 (10)	4 (10)	0

## Discussion

This is the first report of efficacy and safety data for crizotinib among Chinese patients. In the present study, we included patients with poor PS after extensive previous treatment. Our data confirmed that crizotinib was highly effective in Chinese patients, with poor PS patients deriving symptomatic relief. Twenty-eight patients (70% of 40 patients) were classed as having achieved clinical benefit (PR or SD), with a median duration of clinical benefit of 24 (4–60) weeks. Seventeen patients died during or after the study. The median OS was 40 weeks (95% CI 38.6 to 49.3 weeks).

In vitro and in vivo studies have validated ROS1 and ALK as useful targets in NSCLC [9]. The rate of ALK rearrangement is low among NSCLC patients, and there is no difference in prevalence between Asian and non-Asian patients. Patients with wild-type EGFR in advanced stage NSCLC have few treatment options if disease progression occurs after first- or second-line chemotherapy. The approval of crizotinib provides an option for ALK-positive NSCLC patients. In the PROFILE 1007 study of previously treated patients with ALK-positive, advanced-stage NSCLC, crizotinib more than doubled the median PFS compared with standard chemotherapy. In our study, 34 of the 40 patients had received 2–3 lines of chemotherapy, and 22 of them derived clinical benefit (PR or SD) from crizotinib treatment. Our data did not show a significant difference in response rates depending on the number of previous treatment lines (data not shown).

Among 9 patients who developed PD in brain metastases, 6 patients had new lesions in the brain. Crizotinib seems to have poor blood-brain barrier penetration, reducing the anticancer effect of this drug in metastatic brain tumors. We suspect that crizotinib is subject to the same mechanism as EGFR TKIs, where a low CSF-to-plasma ratio occurs in patients who continue to have systemic disease control with gefitinib or erlotinib but display progression or new-onset CNS disease [10]. Further studies are needed to explore the pharmacokinetic properties of crizotinib. Currently, for patients with new or uncontrolled brain metastases but systemic disease control with crizotinib, we suggest radiotherapy to the brain while taking crizotinib concurrently. Further studies are warranted to confirm this strategy.

Previous studies only enrolled patients with good PS (ECOG 0–2). However, most patients' performance status worsens after intense chemotherapy. Oral drugs such as crizotinib are a reasonable choice of treatment for such patients. Our study included 16 patients with PS 3 or 4 and short life expectancies. Dramatic efficacy was noted in some patients shortly after the beginning of treatment. All 16 patients experienced symptom relief, for example, in pain and anorexia, which can seriously affect QOL. Other patients also benefit in terms of symptom relief. As a result, a significant overall improvement in baseline QOL was observed in patients treated with crizotinib.

The adverse events observed in patients treated with crizotinib in this retrospective study were generally consistent with the drug's known adverse event (AE) profile. The most common AEs were vomiting (47.5%), elevated liver transaminases (40%), vision disorders (27.5%), and diarrhea (5%). The most common Grade 3/4 AEs included elevated transaminases (10%) and vomiting (5%). Only a few patients needed dose modifications or treatment breaks.

## Conclusion

Our data are consistent with previously reported efficacy and safety findings and further support the use of crizotinib in patients with ALK-positive lung cancer.
